# Barcoding reveals complex clonal behavior in patient-derived xenografts of metastatic triple negative breast cancer

**DOI:** 10.1038/s41467-019-08595-2

**Published:** 2019-02-15

**Authors:** D. Merino, T. S. Weber, A. Serrano, F. Vaillant, K. Liu, B. Pal, L. Di Stefano, J. Schreuder, D. Lin, Y. Chen, M. L. Asselin-Labat, T. N. Schumacher, D. Cameron, G. K. Smyth, A. T. Papenfuss, G. J. Lindeman, J. E. Visvader, S. H. Naik

**Affiliations:** 1 ACRF Stem Cells and Cancer Division, The Walter and Eliza Hall Institute of Medical Research, Parkville, VIC 3052 Australia; 2 Department of Medical Biology, The University of Melbourne, Melbourne, VIC 3010 Australia; 3 Olivia Newton-John Cancer Research Institute, Heidelberg, VIC 3084 Australia; 4 School of Cancer Medicine, La Trobe University, Bundoora, VIC 3086 Australia; 5 Molecular Medicine Division, The Walter and Eliza Hall Institute of Medical Research, Parkville, VIC 3052 Australia; 6 Bioinformatics Division, The Walter and Eliza Hall Institute of Medical Research, Parkville, VIC 3052 Australia; 7 Immunology Division, The Walter and Eliza Hall Institute of Medical Research, Parkville, VIC 3052 Australia; 8 Division of Molecular Oncology & Immunology, Netherlands Cancer Institute, Amsterdam, 1066 CX The Netherlands; 9 School of Mathematics and Statistics, The University of Melbourne, Melbourne, VIC 3010 Australia; 10 Peter MacCallum Cancer Centre, Melbourne, VIC 3000 Australia; 11 Sir Peter MacCallum Department of Oncology, University of Melbourne, Melbourne, VIC 3010 Australia; 12 Department of Medical Oncology, The Peter MacCallum Cancer Centre, Melbourne, VIC 3000 Australia; 13 Department of Medicine, The University of Melbourne, Melbourne, VIC 3010 Australia; 14 Parkville Familial Cancer Centre, The Royal Melbourne Hospital and Peter MacCallum Cancer Centre, Parkville, VIC 3050 Australia

**Keywords:** Genetic techniques, Breast cancer, Tumour heterogeneity

## Abstract

Primary triple negative breast cancers (TNBC) are prone to dissemination but sub-clonal relationships between tumors and resulting metastases are poorly understood. Here we use cellular barcoding of two treatment-naïve TNBC patient-derived xenografts (PDXs) to track the spatio-temporal fate of thousands of barcoded clones in primary tumors, and their metastases. Tumor resection had a major impact on reducing clonal diversity in secondary sites, indicating that most disseminated tumor cells lacked the capacity to ‘seed’, hence originated from ‘shedders’ that did not persist. The few clones that continued to grow after resection i.e. ‘seeders’, did not correlate in frequency with their parental clones in primary tumors. Cisplatin treatment of one *BRCA1*-mutated PDX model to non-palpable levels had a surprisingly minor impact on clonal diversity in the relapsed tumor yet purged 50% of distal clones. Therefore, clonal features of shedding, seeding and drug resistance are important factors to consider for the design of therapeutic strategies.

## Abstract (web summary)

Triple negative breast cancers (TNBC) disseminate and metastasise, but the clonal relationship of metastases to primary tumours is poorly understood. Here, the authors use cellular barcoding of TNBC patient-derived xenografts and track the fate of barcoded clones in primary tumours and their metastases, including after resection or chemotherapy.

## Introduction

Intratumoral heterogeneity is generated through genetic and non-genetic processes that result in a single tumor comprising diverse subclones, which can evolve along complex trajectories^1,2^. The implementation of high-throughput genomic sequencing approaches at the population level, and now single cell level, has demonstrated extensive molecular heterogeneity within solid tumors, including breast cancer^3^. This complexity has direct bearing on our understanding of tumor evolution, metastasis, drug resistance, and the sampling of lesions from patients to identify the most useful therapeutic targets. One caveat of the single-cell genomics approach is that, due to current technical limitations, only a minute fraction of single cells from a whole tumor and distal sites can be sampled. Furthermore, genomic alterations are only one source of tumor heterogeneity, with transcriptome, epigenome and post-translational control mechanisms also recognized as contributing to heterogeneity^4,5^. Critically, a systematic assessment of the growth and metastatic characteristics of individual cells within primary tumors in a patient-derived xenograft (PDX) setting and peripheral organs, and their response to therapeutic intervention, is lacking.

Breast cancer mortality is caused by metastasis; a complex process involving dissemination, deposition, and growth of tumor cells at distant sites^6,7^. Triple negative breast cancer (TNBC), characterized by a lack of expression of HER2 and the hormone receptors estrogen (ER) and progesterone (PR), has a propensity to disseminate to visceral organs including the lungs and liver^8,9^. Studies on paired patient autopsy samples have highlighted heterogeneity between primary tumors and metastases^9–12^. Analyses of primary breast tumors and brain metastases with their counterpart PDX models has indicated that metastatic lesions contain de novo mutations not present in the original tumor, and likely arise from a minor subset of tumor cells^13–15^. Clonal tracking studies using PDX tumors^16,17^ or a mouse mammary tumor cell line^18^ have revealed diverse clonal growth patterns amongst transplanted tumors in mice, while single-cell analysis indicated a stem-cell program linked to metastatic potential^19^.

We sought to assess the clonal diversity within human drug-naïve breast tumors and their resulting metastases, and how this changed with therapeutic intervention. Open questions included: what fraction of clones can disseminate; how reflective are these of the primary tumor; which clones contribute to metastatic disease in different organs; and what is the consequence of therapy to clonal diversity? To this end we utilized breast PDXs, which largely retain the original patient tumor heterogeneity and exhibit reproducible kinetics during secondary xenografting^13–15,20,21^. Moreover, these models enable studies on the behavior of human ‘drug-naïve’ tumors in a physiological and therapeutic context, in contrast to specimen analysis at autopsy, and are thus considered ‘pseudo-primary’ tumor models. Rather than assess a subset of sampled cells, e.g. through single cell genomic means, we sought to capture the majority of tumor biomass and tumor cells at distal sites, and examine their heterogeneity in an unbiased fashion. Distal sites include cells that have shed from the primary tumor and are found in the blood stream as circulating tumor cells (CTCs), or in other distal organs (e.g. lung, bone marrow, ovaries, and kidney) where they accumulate as disseminated tumor cells (DTCs). We therefore utilized cellular barcoding, which allows robust assessment of clonal diversity and numbers at high resolution and depth, including confident detection of small clones of, e.g. 5–10 cells amongst millions^22^. Our findings provide insights into the spatio-temporal diversity of clones in metastatic disease, and in response to therapy, with implications for the diagnosis, monitoring, and treatment of patients.

## Results

### Engraftment and clonal growth of transplanted barcoded PDXs

We examined 15 TNBC PDXs and found through detailed analyses of multiple organs that three gave rise to macroscopic metastases. These three TNBC tumor models, PDX-110 (*BRCA1*-mutated), PDX-322, and PDX-744 were derived from samples taken at the time of surgery from drug-naïve patients. All three tumors were transplanted and gave rise to primary tumors and metastases (Supplementary Table 1 and Supplementary Figure 1A and B) and were intermediate in their growth kinetics amongst our PDX bank and comparable to those reported by others^23^. The clinical, histopathological, and molecular features of these tumors have been previously described^24,25^. PDX-110, PDX-322, and PDX-744 metastasized to multiple sites including lungs and liver (Supplementary Figure 1B), while PDX-110 also colonized the brain and PDX-322 seeded lymph nodes and adrenal glands, mimicking metastatic sites observed in the corresponding patients (Supplementary Table 1). Metastases were confirmed to be epithelial in origin by immunostaining for keratin expression (Supplementary Figure 1C).

**Fig. 1:**
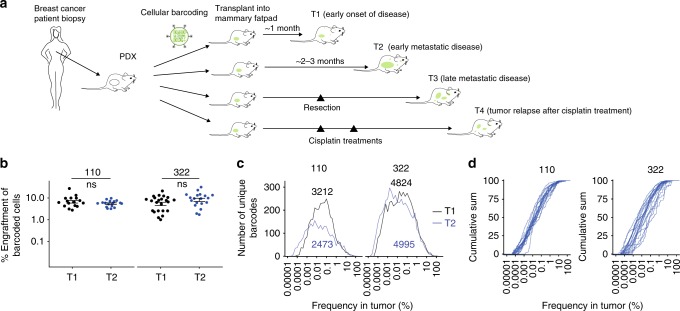
Experimental overview, and spatio-temporal clonal heterogeneity in pseudo-primary tumors. **a** Cellular barcoding of PDXs transplanted into NSG mice and interrogated at different time points and under various conditions. Primary tumors and/or metastases were collected at early stage (T1; 46 days for PDX-110 and 41 days for PDX-322), tumors at ethical-endpoint (T2; 95 days for PDX-110, 58 days for PDX-322), late metastatic disease after tumor resection (T3; 71 days after tumor resection for PDX-110, 84 days for PDX-322), and tumor relapse after chemotherapy (T4; for PDX-110, 110 days for 1 cycle Cisplatin, 130 days for 2 cycles). Times of harvest are the mean. **b** Engraftment efficiencies of PDX-110 and PDX-322 assessed at T1 and T2 (Welch two-sided two sample *t*-test, ns: non-significant, *p* > 0.05). **c** Frequency distribution of clones at T1 and T2 pooled over several mice (PDX-110: 16 mice at T1 from two independent experiments, 17 mice at T2 from two independent experiments, PDX-322: 23 mice at T1 from four independent experiments, 19 mice at T2 from three independent experiments). Total numbers of barcodes used for this analysis are indicated next to the respective distributions. **d** Cumulative size distribution of barcoded clones in primary tumor for PDX-110 and PDX-322 (PDX-110: 16 mice at T1, 17 mice at T2, PDX-322: 23 mice at T1, 19 mice at T2)

We next utilized cellular barcoding to gain insight into the engraftment and growth characteristics of individual cells within early passage PDX tumors^26^. Cell suspensions prepared from tumor xenografts (XP3) were barcode-labeled (Fig. 1a and Methods), cultured for 48 h, sorted by flow cytometry for barcode integration (i.e. GFP expression), and then transplanted into the mammary fat pads of NSG (NOD/SCID/IL2Rγ_c_^-/-^) immunodeficient mice. Different numbers of barcoded GFP^+^ cells (between 500 and 10,000) were sorted 2500 cells per mammary fat pad were chosen for all subsequent experiments to maximize barcode number given our barcode library size, while avoiding repeat use of barcodes^26^. Barcode composition was assessed by PCR and sequencing from tumors, CTCs, and distal sites of metastasis at different stages of disease progression (Fig. 1a). These included early stage disease (T1), tumors at ethical-endpoint (T2), late metastatic disease after tumor resection (T3), and tumor relapse after chemotherapy (T4).

Quantification of the number of barcodes in primary tumors at T1 and T2 demonstrated an engraftment efficiency of ~10% for PDX-110 and PDX-322, respectively (Fig. 1b), and ~1% for PDX-744 (Supplementary Figure 1D). The range of observed efficiencies was variable between PDXs, consistent with previous findings^27^.

Notably, the average number of barcoded clones remained unchanged throughout the course of primary tumor growth for PDX-110 and PDX-322 (Fig. 1b, T1 vs. T2, *p*-values based on Welch two sample *t*-test: 0.17 (PDX-110) and 0.14 (PDX-322)), indicating negligible clonal extinction occurred once the tumor was established. A broad clone size distribution was evident for all tumors (Fig. 1c, d; Supplementary Figure 1E, F), consisting of both major and minor clonal populations spanning up to a million-fold difference in frequency, in tumors of up to 10–100 million cells. Notably, the appreciation of this level of diversity would not be achieveable with current single cell genomic strategies. Across replicates, a more consistent distribution of clone sizes was observed for PDX-110 compared to PDX-322 and to a larger extent, PDX-744 (Fig. 1d; Supplementary Figure 1F, average inter-animal *p*-values using two-sample K–S test: 0.55 ± 0.21 (mean ± SD, PDX-110) and 0.15 ± 0.09 (mean ± SD, PDX-322)). For PDX-744, this partly reflected the small number of clones that engrafted (Supplementary Figure 1E).

Due to the low degree of engrafted clones in PDX-744, we focused on PDX-110 and PDX-322 for our longitudinal analysis of metastatic properties. Overall, the broad distribution in clone sizes amongst the different PDXs indicates that clones differ greatly in their relative expansion from single cells, likely reflecting non-mutually exclusive factors including the molecular nature of individual clones, stochastic effects, and/or the influence of the microenvironment.

We assessed the genomic heterogeneity of PDX-110 and PDX-322 in early passage xenografts after their establishment and observed substantial genetic alterations (Supplementary Figure 2). Analysis of copy number based on scRNA-seq data from 9939 sorted tumor cells for PDX-110 and 14,463 tumor cells for PDX-322 using inferCNV^28^ revealed shared sub-clonal, large-scale copy number gains, and losses across the genome in both PDX models. While not able to directly link barcoded clones with genomic and transcriptional clonal heterogeneity, this analysis excludes the possibility that the observed heterogeneity was solely due to random sampling or clonal drift of cells derived from a genetically homogeneous PDX.

### Clonal mosaicism of primary tumors

**Fig. 2:**
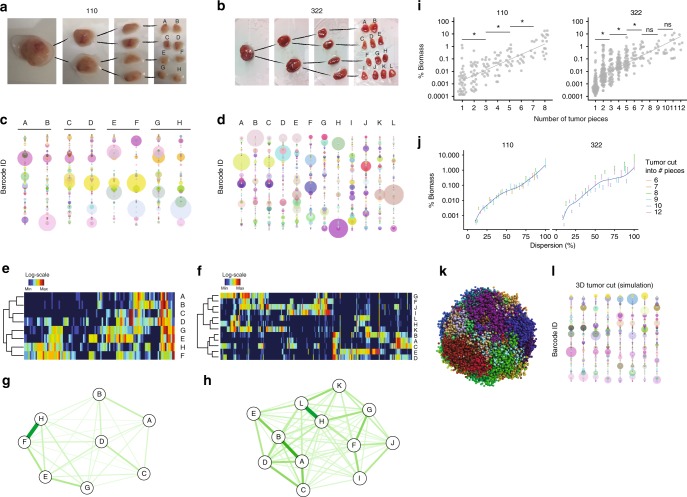
Clonal mosaicism in three dimensions. Example of tumor cut into **a** Eight equal pieces for PDX-110 and **b** 12 equal pieces for PDX-322. **c**, **d** Representative bubble plot of clonal relationships between tumor pieces shown in **a** and **b**, respectively. Each barcode is represented by a dot of a given color, the size of the dot correlates with the number of reads after sequencing. The color codes are not related between different mice. **e**, **f** Heatmap representation with hierarchical clustering of clonal relationships for the example shown in **a** and **b**, with barcodes in columns and tumor pieces in rows. **g**, **h** Force-directed graph-based spatial reconstruction of tumor using Hellinger distance between barcode distributions in pieces. **i** Contribution to tumor biomass as a function of the number of pieces a given clone is detected for **a** and **b**. Welch two-sided two sample *t*-test, *p < 0.05, ns = non significant. **j** Relationship of % biomass and dispersion (defined as the number of pieces a barcode is detected scaled by the total number of pieces per tumor) pooled over several mice (PDX-110: 17 mice, PDX-322, 19 mice). **k** Simulation and visualization of three-dimensional tumor growth, initiated with 200 barcoded PDX cells. Each dot is a cell, and colors indicate cells with different barcodes. **l** Bubble plot of a virtual tumor cut into eight equal pieces

Tissue sampling, or biopsy, is a common diagnostic procedure, but the extent to which a small fragment reflects the composition of the entire tumor is an open question. To address the clonal and spatial heterogeneity within PDX-110 and PDX-322, barcoded tumors were collected at T2 and dissected into pieces of similar size (2–15 pieces of around ~75 mm^3^, depending on tumor size) (Fig. 2a, b, representative example). The number and frequency of barcodes of the entire organ or tumor biomass were determined by sequencing (Fig. 2c–f, representative example) exhibiting robust, sensitive, and representative (i.e. non-random) detection (Supplementary Figure 1H). Each piece of primary tumor contained a unique profile of barcodes, both in their composition and relative abundance. This was visualized in two ways and illustrated by two representative examples for PDX-110 and PDX-322. First, using ‘bubble plots’, where barcodes were distributed along the *y*-axis, assigned a unique color, and their bubble size scaled according to clonal abundance (Fig. 2c, d). This allowed ease of comparison along the *x*-axis of a given barcode clone across multiple tumor pieces, within one mouse. Second, heatmaps allowed full appreciation of the number of clones, and allowed hierachical clustering between tumor pieces (Fig. 2e, f). Combined, we observed that adjacent pieces typically shared some barcodes, while other barcodes were distributed throughout the tumor. Using a force-directed graph based on Hellinger distance (Fig. 2g, h), we spatially reconstructed the tumor pieces in two dimensions, which largely aligned with the known positional relationships of tumor pieces in Fig. 2a, b. Thus, barcode diversity and composition were heterogeneous, and fit with a model in which cells grow in spatial ‘patches’ in PDXs, recapitulating clonal heterogeneity in patient tumors^29–33^.

As a general trend, the number of pieces in which a barcode was detected increased with its biomass in the total tumor (Fig. 2i). As the exception to the rule, however, we also noted some small clones (<0.1% of tumor biomass) were highly dispersed throughout the tumor, whereas some large clones (>1%) were localized to one or a few pieces (Fig. 2i). By pooling over multiple mice and measuring this ‘dispersion’, defined as the number of pieces a barcode was detected scaled by the total number of pieces per tumor, confirmed a consistent positive correlation of clone size with dispersion (Fig. 2j, Supplementary Figure 1G). To better visualize the spatial heterogeneity that may underlie the patterns that we observe in Fig. 2c, d, we minimally adapted a modeling framework of 3D cancer growth^34^ to include barcoding (Fig. 2k), and the virtual cutting of this model closely mimicked our data (Fig. 2l).

Through whole tumor assessment performed here, our results suggest that tumor biopsies are unlikely to capture the full clonal complexity of TNBC tumors due to clonal mosaicism, in agreement with previous studies using intravital imaging^35,36^ and genomic sequencing^29–33^.

### Clonal relationships between primary tumors, CTCs, and DTCs

A central clinical question is the utility of CTCs as liquid biopsies^37,38^. For this to be informative of the composition of primary tumor, the majority of subclones within a tumor must have the ability to shed progeny into circulation and must persist sufficiently long in the bloodstream. Clones could conceivably differ in respect to these processes, including their propensity to shed, their rate of shedding, and time prior to clearance, such that the frequency of CTCs may differ in their representation of the primary tumor. To address how representative CTCs and lung DTCs were of the primary tumor at a clonal level, we utilized our models to compare these tissues at T2, where tumors were at maximum ethical size in order to track putative shedding clones detectable in distal organs (Fig. 1a).

**Fig. 3:**
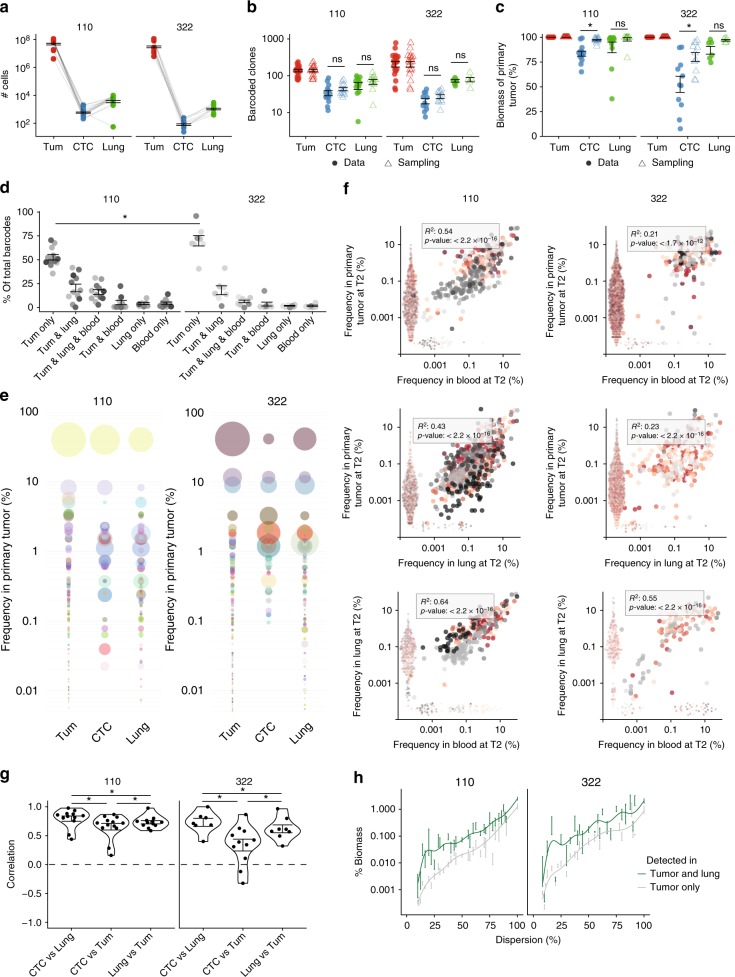
Clonal relationships between primary tumor and distal sites. **a** Number of barcoded cells detected in tumor (approximation), blood, and lung at T2. Bars represent means ± SEM. *p < 0.05. ns = non-significant. Number of cells in tumor are estimated from tumor volume (see Methods). Number of cells in blood and lung correspond to GFP^+^ cells FACS-sorted from whole blood or lung. **b** Number of clones detected in tumor, blood, and lung at T2. Bars represent means ± SEM. Welch two-sided two sample *t*-test, *p < 0.05, ns = non-significant. **c** Percentage of the biomass of primary tumor represented in blood and lung at T2. Bars represent means ± SEM *p < 0.05. Welch two-sided two sample *t*-test, *p < 0.05, ns = non-significant. For **b** and **c**, filled dots represent data and empty triangles represent the results obtained by simulation. **d** Clonal overlap between tumor, blood, and lung. **e** Representative bubble plots of clonal relationships between primary tumors, CTCs, and lung DTCs. **f** Scatter plots of clonal frequencies of tumor versus blood, tumor versus lung, and lung versus blood. Dots on the axis are barcodes only found in one tissue, and dispersed for ease of visualization. Different shades of red and gray indicate different mice (*n* = 19 mice for PDX-110 from three independent experiments and *n* = 13 mice for PDX-322 from three independent experiments). Inset gives adjusted *R*^2^ and *p*-values using the *F*-test for linear regression on log-transformed frequencies of barcodes detected in both tissues. **g** Correlations in log-transformed clonal frequencies detected in two respective tissues. Each dot represents a mouse (*n* = 19 mice for PDX-110 from three independent experiments and *n* = 13 mice for PDX-322 from three independent experiments). Bars represent mean ± SEM. Welch two-sided two sample *t*-test, *p < 0.05, ns = non-significant. **h** Relationship of % biomass and dispersion for clones detected and not detected in lung (mean ± SEM)

For analysis of cells at distal sites, we focused on PDX-110 and PDX-322 due to their greater clonal diversity. The numbers of CTCs and lung DTCs were ~10^5^-fold lower compared to the primary tumor (Fig. 3a). Despite this large difference in total cell number, the reduction in clone number compared to primary tumor was, although significant (Welch two-sample *t*-test), relatively marginal (e.g. 2.3-fold (PDX-110) and 3.5-fold (PDX-322) reduction between tumor and lung) for PDX-110 and PDX-322 (Fig. 3b). As a measure of how reflective CTCs and lung DTCs were of the primary tumor, we determined the proportion of their parental barcoded clones in the primary tumor biomass (see Methods). We established that parental clones of those detected in CTCs contributed to 80% of biomass in PDX-110 tumors, suggesting that most of these represented larger clones. For PDX-322, however, there was a broad range from 5% to 90% in different mice (Fig. 3c).

Many clones were detected only in the tumor: on average, 50% of clones for PDX-110 and 70% for PDX-322, which was significantly different, presumably through differential rates in either shedding and/or clearance. Clones could also be detected in lung and/or blood at the time of harvest (Fig. 3d). This implied that a significant proportion of clones shed circulating progeny into blood, where they serve as in-transit cells with the potential to accumulate in lungs and other distal sites^39^. Interestingly, in CTCs we observed for PDX-110 a significant increase (*p*-value: 0.0053, Welch two sample *t*-test) in barcode numbers between T1 and T2 (Supplementary Figure 3A), but not for PDX-322. Clonal distribution at both timepoints are shown in Supplementary Figure 3B.

To better understand the dependency between the volume of blood that is analyzed and the number of detected barcodes, we split the terminal blood sample into 10 smaller (~80 µl) samples and assessed barcode diversity in each one. By incorporating fewer and fewer samples in the analysis we observed a clear drop in detected barcodes (Supplementary Figure 3C), but a less marked change in representative biomass (Supplementary Figure 3D), indicating that some clones are not detectable if blood volumes are too small, but those barcodes that remain in the smallest sample we tested (80 µl) still represented 50% of tumor biomass. How this relates to a patient setting and CTC assays where a much smaller relative amount of blood is drawn (10 ml out of 5 l) is a highly relevant question. The answer will be highly dependent on the total number of CTCs, the way in which the cells are isolated and their clonal diversity in each patient. Overall, these findings are important as they highlight that CTCs are likely to be unpredictable in the degree to which they represent clones that comprise the majority of primary tumor, with potential implications for the use of CTCs in diagnosis. With our methodology, we could not determine whether this represents a differential ability to shed, to be cleared by the host, and/or die when outside the tumor environment.

Hypothetically, if all clones had an equal ability to shed, then their chance of detection would be proportional to (i) their clone size in the primary tumor and (ii) the sensitivity of detecting limited number of cells in blood and lung. To test this, we performed in silico subsampling using the number of cells originally sorted from blood and lung (Fig. 3b, c) and based on the barcode distribution measured in the tumor. We found that the number of clones and biomass representated in primary tumor in the sampled and experimental data was comparable for both PDX models (Fig. 3b, c, triangles), with the exception of CTC, for which the biomass was significantly lower than expected from the above hypothesis (*p*-values: 0.008 for PDX-110 and 0.00018 for PDX-322 using Welch two-sample *t*-test).

We then determined which barcodes were detected in tumor, and/or lung, and/or blood to compare the relative frequencies and correlations. While barcoded clones differed in their relative frequency within an organ, they generally correlated in frequency between organs (Fig. 3e–g). Correlation between CTCs and lung was significantly higher than the correlation between CTCs and tumor, or lung and tumor (Fig. 3g), in line with CTCs being trapped in the highly vascularized lung. Compared to in silico subsampling, however, the correlation was less than expected (Supplementary Figure 3E), and particularly reduced for PDX-322 (Fig. 3f, g), indicating that our null hypothesis of direct proportional sampling is likely to be over simplified. In summary, large clones in primary tumors gave rise to higher numbers of detectable cells in peripheral organs, although some clones were over-represented and under-represented. Clones detected in lung did statistically contribute more (in average ~9-fold (PDX-110) and 5-fold (PDX-322)) to tumor biomass (Wilcoxon rank sum test with continuity correction, *p*-value: <2.2^−16^), regardless of their dispersion (Fig. 3h).

### Distinction between shedders and seeders

**Fig. 4:**
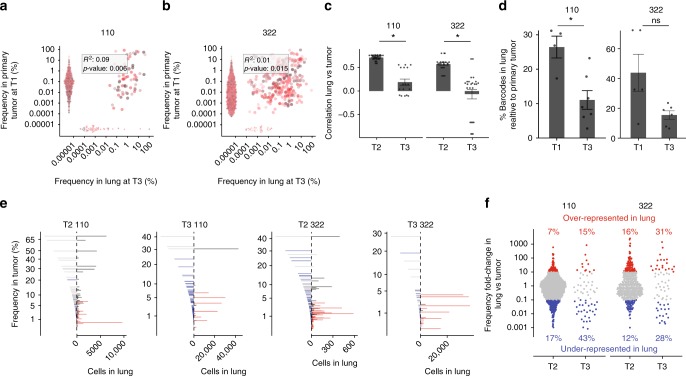
Characteristics of metastatic ‘seeder’ clones after tumor resection. Clonal relationships between resected primary tumor at T1 vs. metastatic lung disease at T3 in **a** PDX-110 and **b** PDX-322. Each color represents the clones from one mouse of a total of seven mice for PDX-110 from two independent experiments, *n* = 9 mice for PDX-322 from three independent experiments. Inset gives adjusted *R*^2^ and *p*-values using the *F*-test for linear regression on log-transformed frequencies of barcodes detected in both tissues. **c** Correlation of clonal relationship between indicated organs based on **a** and **b** between distal tissues at T3 compared to resected primary tumor at T1. Data represent 20 mice for PDX-110 from two independent experiments and 23 mice for PDX-322 from four independent experiments (mean ± SEM). **d** Change in the proportion of barcodes detected in lung prior to (T1) and after (T3) tumor resection in the indicated PDX (mean ± SEM). **e** Visualization of the clonal frequency of barcodes in primary tumor by bar length (left side of dotted line) and ordered by frequency along the *y*-axis (represented on square-root scale to highlight dominant clones), with the corresponding number of cells of the same clone estimated in lungs (right sides of dotted line). Blue bars indicate clones that were under-represented (<10-fold compared to frequency in primary tumor), and red bars indicate over-represented in lung (>10-fold compared to frequency in primary tumor). **f** Ratio of frequency in primary tumor compared to lung for all barcodes from all mice detected in both organs, with a cut-off for clones 10-fold over-represented (red) and 10-fold under-represented (blue), with indicated proportions of red and blue barcoded clones when tumor was present and large (T2), and at late metastatic disease after tumor resection (T3)

In order to model advanced metastatic disease, primary tumors were resected (once they reached 150 mm^3^), and mice euthanized after they developed symptomatic metastatic disease (~1–2 months post-resection at T3) from pre-existing metastatic tumor cells (Fig. 1a). Surprisingly, we found that the correlation of barcode frequency between lungs and primary tumors was significantly reduced in both PDX-110 and PDX-322 (Fig. 4a–c). There were two main aspects to these findings: firstly, we identified a large drop in barcode numbers between the time of resection (T1) and late metastatic disease (T3) (Fig. 4d). This indicated that many clones in primary tumors were able to disseminate progeny into blood and lung (‘shedders’), but more than half extinguished at distal sites once primary tumor was removed. This could be explained if these clones were merely ‘shedding’ cells into different organs (at a rate proportional to their size in tumor), while lacking the ability to grow. On the other hand, those that survive and grow (i.e. ‘seeders’) established stable metastases outside the primary tumor, and this could have occurred either prior or post resection. Secondly, there was a larger disparity (i.e. loss of correlation) in frequency between primary tumor at T1 and lung at T3. Relatively minor clones in primary tumor contributed to most of metastatic burden, while major clones disappeared or diminished significantly (Fig. 4a, b, e). When barcode frequency was compared as a fold change (Fig. 4f), the number of clones that differed from their expected frequency (i.e. the frequency in primary tumor) by more than 10-fold almost doubled in proportion.

Taken together, our results indicate that clone size in primary tumor at the time of resection (T1) is neither predictive for the presence or absence of clones in lung at late metastatic disease, nor predictive of the metastatic contribution of these clones to lung.

### Seeder clones are a smaller subset of shedding clones

**Fig. 5:**
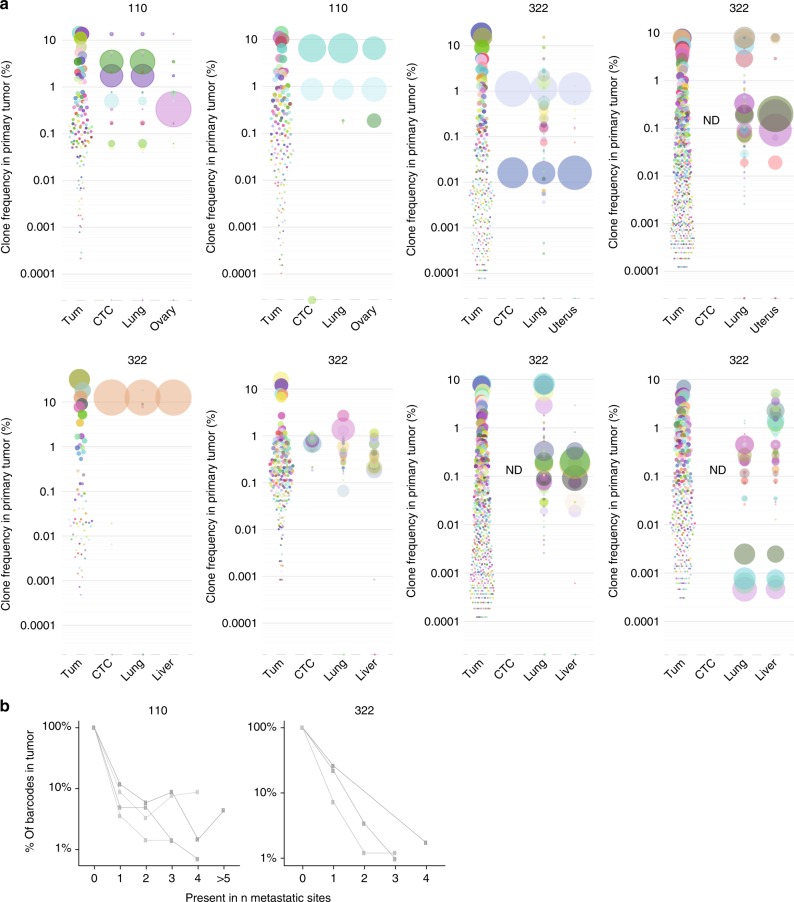
Properties of multi-organ metastases. **a** Individual mouse examples of clonal relationships between indicated organs from PDX-110 and -322. Each barcode is indicated by a color for individual mice. The color codes are not conserved between different mice. **b** Number of clones that are found in none (i.e. in tumor only) or one or more metastatic sites for 4 (PDX-110) or 3 (PDX-322) individual mice (each mouse represented by a line)

Whether the capacity to ‘seed’ is related to the environment of the distal site, or an intrinsic property of the clone is not thoroughly understood. If the capacity to metastasize were an intrinsic property of a clone, not excluding any niche-specific factors, then one might expect their detection in multiple organs. Indeed, we found that several barcoded clones in both models could be detected in three or more organs, whereas other clones were only present in one (Fig. 5a, b; Supplementary Figure 4). Overall, relatively few clones were able to stably metastasize relative to the number detected in primary tumor and, as observed earlier (Fig. 4), their prevalence did not correlate with clone size in the primary tumor. Further studies will be needed to address the mechanisms underlying these observations.

### Impact of chemotherapy on clonal diversity

Given that heterogeneity represents an important obstacle for patient therapy, we next determined the impact of chemotherapy on tumor heterogeneity, and the clonal repertoire associated with local and distal relapse of disease. Our hypothesis was that tumor relapse following a near complete pathological response, in which tumors shrunk to non-palpable levels, would select for a small number of refractory clones. We selected PDX-110 (*BRCA1*-mutated; Supplementary Table 1) to examine the impact of chemotherapy on tumor heterogeneity, as this PDX was highly responsive to cisplatin therapy. PDX-322 did not respond well to cisplatin nor a range of alternative agents tested (e.g. taxane) (data not shown). As tumors never reached non-palpable levels, this model was not suitable for testing our hypothesis.

**Fig. 6:**
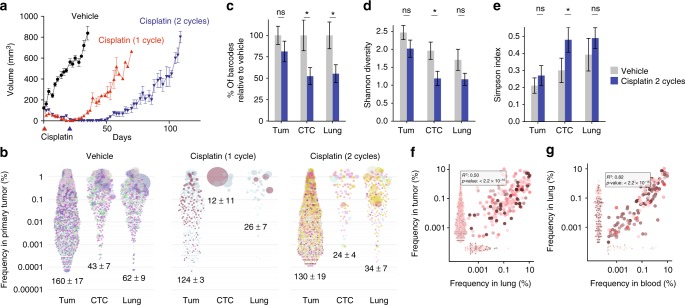
The effect of cisplatin on clonal diversity. **a** Average volume of tumors from PDX-110 treated with vehicle, or cisplatin (6 mg/kg, treatment time indicated by a triangle at the bottom for either 1 or 2 cycles). Tumor volume from 12 mice per group (mean ± SEM). **b** Bubble plot of clonal relationships between indicated tissues of all mice, with indicated average barcode number per mouse (mean ± SEM) in tumor, CTC, and lung. Colors indicate different mice. **c** Percentage of barcoded clones in vehicle or cisplatin treatment group at tumor relapse relative to vehicle-treated controls in indicated tissues (shown is mean ± SEM, ns: *t*-test, not significant, *p < 0.05). **d** Shannon diversity and **e** Simpson index of barcode distribution in different organs in the treated and untreated group. Scatter plots showing the correlation between the frequencies of barcoded clones in primary tumor versus **f** lung (*n* = 10 mice) and **g** blood and lung (*n* = 9 mice). Inset indicates adjusted *R*^2^ and *p*-values using the *F*-test for linear regression on log-transformed frequencies of barcodes detected in both tissues

When the primary tumors reached 150 mm^3^, barcoded PDX-110 was treated with cisplatin (Fig. 1a), which is commonly used for the treatment of *BRCA1*-associated TNBC. Despite a profound reduction in primary tumor biomass after one or two cycles (Fig. 6a) of treatment to non-palpable levels, tumors relapsed at both local and distal sites. Surprisingly, on comparison with vehicle, we observed only a slight decrease in the number of barcoded clones that constituted the bulk of relapsed tumor biomass (not significant) (Fig. 6b, pooled over several mice, Fig. 6c, Supplementary Figure 5). Rather than selecting for a few refractory clones, the majority of barcoded clones (~80%) survived cisplatin treatment and regrew in primary tumor, with 50% remaining detectable in the periphery (Fig. 6c). This rejected our hypothesis and was non-intuitive considering tumor biomass depleted rapidly within ~20 days, with a 25–30 days delay before tumors relapsed. It indicates that ~80% of drug-naïve barcoded clones, large or small, can harbor at least one cell that was refractory to two doses of cisplatin.

Three months post-chemotherapy with two cycles of cisplatin (T4), we used two measures to assess changes in clonal diversity; Shannon diversity and the Simpson index. In both cases, the parameters did not change significantly for relapsed tumors, and only slightly changed for CTCs and lung (significant for CTCs) (Fig. 6d, e). However, the clones found in lungs and CTCs still correlated with each other in terms of frequency and with the primary tumor (Fig. 6f, g). Collectively, these data show that chemotherapy had a remarkably minor effect on the extinction of barcoded clones within PDX-110 primary tumors, despite a more profound effect on the number of barcodes detected in the periphery.

## Discussion

Here we combined cellular barcoding with PDX models for high-resolution clonal assessment of metastatic drug-naïve TNBC, both longitudinally, under different conditions, and across multiple tissue sites. Such a holistic assessment of the contribution of single barcoded cells to PDX tumors and metastases across all sampled tissues could not be easily achieved through either (i) somatic mutation-based clonal analyses in bulk tumors, which cannot disentangle bona fide minor clones of similar frequency or (ii) assessment of individual tumor cells from treated patients at autopsy^40^, which have already undergone clonal evolution and would potentially require assessment of millions of single cells for a comprehensive analysis. Moreover, the use of PDXs allowed comparison of metastatic disease in different temporal, spatial and treatment scenarios and in a drug-naïve context. Rather than analysis of genomic heterogeneity in tumors, for which there is a large body of accumulating data, we focused on a functional read-out of barcoded clones that revealed their complex clonal behavior across time, space, and in response to treatment.

We demonstrate inter-tumoral and intra-tumoral heterogeneity that includes unique features of barcoded clones to differentially (a) grow in primary tumors, (b) ‘shed’ into circulation, (c) ‘seed’ distal organs following removal of the parental clone in the primary tumor, and (d) contribute to relapse after chemotherapy. Importantly, our findings indicate that while CTCs can be reflective of the bulk of primary tumor biomass, most are shedders that can be sustained by continuous supply from the primary tumor but do not seed to generate metastases. This finding is consistent with previous genomic studies that have elegantly shown that early DTCs are more heterogeneous than larger metastatic lesions^7,41^, due to the selection of the ‘fittest’ clones. As a result, the diagnostic utility of CTCs to predict metastatic disease may be limited. Moreover, PDXs themselves can differ widely: particularly low engraftment was observed for PDX-744, while PDX-322 and PDX-110 were very heterogeneous in terms of their properties of clone size, shedding, seeding, and the relative clonal frequency between metastases and distal sites, even between mice.

Another important feature we observed was the multi-organ metastatic capacity of very few clones, providing a framework to now test whether this is related to an intrinsic molecular property of rare clones for multi-organ metastatic capability (<10%). Therefore, we promulgate the importance of stratifying clones functionally into shedders and seeders to conceptualize the biology underlying metastatic cancer. The molecular or stochastic etiology of these mechanisms, e.g. whether clone size is an intrinsic property of barcoded single cells or results from stochastic clonal drift remains to determined. While this study has focussed on the importance of relating barcoded clone relationships between the primary tumor and the periphery, it also creates a framework for future work on the tracking of clonal features.

Interestingly, most clones (~80%) survived chemotherapy in a PDX model that showed a significant tumor remission then relapse. This is in contrast to previous findings for a variety of cancers^1,16,42,43^ and cell lines^44,45^ treated with chemotherapy, where most clones were extinguished, and after tumor resection in this study, where the majority of clones at distal sites were cleared. This could be explained by the higher sensitivity of clone detection through cellular barcoding compared to previous studies, but further investigations are needed. Moreover, testing other PDXs for their responsiveness to chemotherapy will be required to understand whether this is a generalizable feature. These findings in this PDX model can be explained if at least one cell in ~80% all treatment-naïve clones survived chemotherapy. This could either indicate that those drug-naïve clones were intrinsically molecularly resistant to chemotherapy compared to the ~20% of clones or, more likely, that resistance was acquired stochastically in a fraction of cells from most clones following chemotherapy, as most recently exemplified in an in vitro study^45^.

In addition, single cell DNA and RNA sequencing prior and post neoadjuvant chemotherapy in patients with TNBC suggested that two models of clonal evolution may co-exist in chemo-resistance: adaptive and acquired chemoresistance^43^. However, studying thousands of single cells present in biopsies or larger pieces of tumor at surgery (a minute fraction of all tumor cells) would largely fail to detect minor clones present in the original or residual tumor in patients.

Whether these features of clonal cancer biology are consistent with real patient scenarios, and whether the immune system (absent in NSG mice) exerts an influence needs to be determined. Furthermore, in patients, tumors can disseminate early during the transformation^46^ and can be subjected to a genomic ‘parallel progression’ during the course of their disease^47^. Currently, it is not clear whether any of these molecular features of heterogeneity overlap with the properties we describe here for barcoded clones. The development of new technologies enabling clone tracking, together with genomic, transcriptomic, and epigenetic analyses, may provide a more complete picture of the mechanisms governing tumor evolution in PDXs and patients in the future. Cellular barcoding together with the analysis of clonal relationships in metastatic PDXs that reflect patient outcomes paves the way for incorporating clonal information in potential diagnosis or treatment strategies for breast cancer.

## Methods

### PDX establishment

Treatment naïve breast cancer primary tumors were collected after consentment of the patients through the Royal Melbourne Hospital Tissue Bank and the Victorian Cancer Biobank with relevant institutional review board approval. Human ethics were approved by the Walter and Eliza Hall Institute (WEHI) Human Research Ethics Committee. To first establish a line of PDX material, prior to barcoding experiments, cohorts of 3-week-old or 4-week-old female NOD-SCID-IL2Rγ_c_^–/–^ female mice were seeded with single cell suspensions of early passage human breast tumors (passage 3). To establish and maintain the PDXs, tumor cells (150,000–250,000) were resuspended in 10 µl transplantation buffer (50% fetal calf serum, 10% of a 0.04% trypan blue solution and 40% PBS) and growth-factor-reduced Matrigel (BD Biosciences) in a ratio of 3:1, and injected into the cleared mammary fat pads of recipient mice. All animals were bred and maintained according to institutional guidelines and protocols were approved by the WEHI Animal Ethics Committee.

### Cellular barcoding and transplantation

For the cellular barcoding experiments, material from above tumors were collected and digested in 150 U/ml collagenase (Sigma) and 50 U/ml hyaluronidase (Sigma) for 1 or 2 h at 37 °C. The suspension was then digested with 0.25% trypsin 1 mM EGTA and 5 mg/ml dispase (Roche Diagnostics) for 1 min at 37 °C and filtered (40 µm). Red blood cells were removed by lysis. Cells were then plated in ultralow attachment plates (Corning) in serum-free mammosphere media containing DMEMF12 with Glutamax (Gibco) supplemented with B27 (Invitrogen), 20 ng/ml EGF (BD Biosciences) and 20 ng/ml bFGF (BD Biosciences), 4 μg/ml heparin (Sigma), 1 µg/ml of Hydrocortisone (Sigma), 5 µg/ml of insulin, and Penicillin–Streptomycin (Gibco). Cells were infected with the barcoded lentivirus library^22^, which contains ~2500 98-bp semi-random DNA barcodes and a GFP reporter. Virus concentration was pre-determined to achieve 10–20% GFP^+^ in PDX cells for 48 h following a 1.5 h spin transduction, which was consistent for PDX-110, PDX-322, and PDX-744. Over 10 transduction batches, the proportion of GFP^+^ cells was 7 ± 4.99% for PDX-110, 12.18 ± 8.13% for PDX-322, and 20.35 ± 7.71% for PDX-744 (mean ± SD). Other studies have previously demonstrated that transduction in the order of 10–20% results in 90–95% of GFP^+^ cells carry a single barcode according to Poisson statistics^26^. To determine the upper bound of multiple integrations, we performed PCR on single cells from a transduction at the higher end (~20% transduction), and found that ~90% of 193 single cells contained a single barcode, which is in line with the expectation through Poisson statistics of 20% transduction. After 48 h, cells were washed and GFP^+^ cells sorted by flow cytometry and injected into a cohort of 3–4-week-old NOD-SCID-IL2Rγ_c_^–/–^ female mice. For each mouse, 2500 barcoded cells were resuspended in 10 µl transplantation buffer and growth-factor-reduced Matrigel (BD Biosciences) in a ratio of 3:1 and injected into the cleared mammary fat pad. Mice were sacrificed at different times (see Fig. 1a), or if their health deteriorated for reasons other than disease progression.

### Tumor and mouse monitoring

Tumor volume was estimated three times weekly using electronic vernier calipers and the formula: (minimum diameter)^2^(maximum diameter)/2. Randomization and tumor measurements were managed using the Study Director software (v 3.0, studylog). Mice were sacrificed when primary tumors reached 150 mm^3^ (T1), at the first measurement where tumor volume exceeded 600 mm^3^ (T2 and T4), or if their health deteriorated due to metastatic disease (T3) or for reasons other than disease progression.

### Tumor resection or cisplatin treatment

Once tumors reached a volume of 150 mm^3^, mice were randomized into surgery and treatment arms. Resection was performed and the tumor was cut into pieces and analyzed as described below. In the absence of local relapse, the organs were collected when the mice succumbed to metastatic disease. For the treatment experiments, mice were treated with one or two cycles of cisplatin (at day 0 and 21; 6 mg/kg) or vehicle (at least 10 mice per arm) and mice were monitored twice per week, until their primary tumors reached ethical endpoint. Tumors, blood, and lungs were collected and barcoded cells (GFP^+^) were sorted prior to analysis. Other organs were fixed in formalin. Cell numbers in primary tumor were estimated assuming 10^6^ cells per mg of tumor weight.

### Organ collection, cell suspension, and FACS-sorting

Blood was collected by terminal end bleed (~800 μl) and the red blood cells were lysed before sorting GFP^+^. Cell suspensions from lungs were prepared by digestion for 1 h at 37 °C with 2 mg/ml of collagenase (Worthington) and 200 U/ml of deoxyribonuclease (Worthington) in 0.2% of d-glucose (Sigma) in DPBS (Gibco). The suspension was then filtrated through a 100 μM cell stainer before red blood cell lysis. Brain pieces were lysed in Viagen buffer (Viagen DirectPCR^®^ with 1:50 Proteinase K 20 mg/ml (Invitrogen) or cell suspensions were obtained by mincing and dissociation of the pieces using collagenase and hyaluronidase digestion for 1 h at 37 °C. Ovaries were analyzed by direct lysis in buffer or mechanical processing and enzymatic processing (collagenase/hyaluronidase digestion for 1–2 h). After red blood cell lysis, GFP^+^ cells were sorted. Bone marrow from flushed femurs was lysed in Viagen buffer. Macrodissected metastases from different organs (liver, uterus, kidney, lymph nodes) were directly lysed in Viagen buffer. For scRNA-seq analysis, after single cell preparation as described^48^, Lin^–^EpCAM^+^ tumor cells were isolated by flow cytometry on the AriaC using anti-CD31 (PE; BD Pharmingen), anti-CD45 (PE; BD Pharmingen), anti-CD235a (PE; BD Pharmingen), anti-EpCAM (CD326; FITC; Stem Cell Technologies), anti-integrin α6 (APC-Cy7; Biolegend), and 7-AAD (live/dead separation).

### Lysis, PCR amplification of the barcodes and sequencing

Pieces of primary tumors or metastases were resuspended in Viagen buffer containing 400 μg/ml proteinase K, before incubation for 1 h at 55 °C and then 30 min at 85 °C. Cell pellets of sorted cells were resuspended in PCR lysis buffer (Viagen) containing 200 mg/ml proteinase K and dispensed into individual wells of a 96-well plate. Plates were covered with a rubber mat and lysed in a thermocycler at 55 °C for 1 h, and then at 85 °C for 30 min to inactivate proteinase K. Samples were only stored at –20 °C after this step. For the first round of PCR, 160 µl of PCR reagents containing TopLib 59-TGCTGCCGTCAACTAGAACA-39 and BotLib primers 59-GATCTCGAATCAGGCGCTTA-39 were added to a 40-µl lysate of all samples. After mixing, 100 µl was transferred to an adjacent empty well before PCR, to provide the technical replicates to assess barcode detection reliability (Supplementary Figure 1H for an example). Plates were sealed and placed in a thermocycler at 94 °C for 5 min, then cycled 30 times at 57.2 °C for 15 s, at 72 °C for 15 s and at 94 °C for 15 s, and then at 72 °C for 10 min. The presence of a 150-bp product was checked for every sample using 2% agarose gel electrophoresis.

In the second round of PCR, a different index primer was used for every sample and technical replicate. To do this, a library of 384 different 82-bp index primers containing unique 8-bp indexes were used (sequences available on request). A mastermix of PCR reagents (24 µl), included a common reverse primer 59-CAAGCAGAAGACGGCATACGAGATTGCTGCCGTCAACTAGAACA-39. Subsequently, 5 μl of up to 384 samples after second round PCR containing different index primers were pooled, and used for cluster generation and sequencing on a Next-seq (Illumina).

### Immunohistochemistry

Tumor and metastatic samples were fixed in 4% paraformaldehyde and embedded in paraffin. Antigen retrieval was performed using pH9 antigen retrieval buffer (DAKO S2375) at 95 °C for 20 min or citrate buffer pH6 at 95 °C for 20 min for CC3. Antibodies against ER (NCL-L-ER-6F11, Novocastra), PR (NCL-L-PGR-312, Novocastra), HER2 (SP3, Spring Bioscience), or pan-cytokeratin (DAKO, Clone AE1/AE3) were used at 4 °C overnight, followed by biotinylated anti-IgG secondary antibodies (Vector Labs). The signal was detected by incubation with ABC Elite (Vector Labs) for 30 min and 3,3′-diaminobenzidine (Dako) for 5 min at room temperature.

### scRNA-seq and copy number analyses

Cell suspensions were prepared as described above for PDX-110 and PDX-322 samples. A 10X Genomics Chromium machine was used to capture 10,000–15,000 single-cells from each tumor into Gel Bead-In-EMulsions (GEMs) and cDNA prepared according to the Single Cell 3′ protocol recommended by the manufacturer and previously described^49^. The final sequencing library contained standard Illumina paired-end constructs flanked by P5 and P7 and was sequenced on an Illumina Nextseq 500 using run parameters described in Single Cell 3′ protocol. Cell Ranger v2.0.0 (10X Genomics) and bcl2fastq v2.19.1.403 were used to generate genewise sequence read counts for each cell.

inferCNV (v0.3)^28^ was used to generate by-cell genome-wide relative copy number estimates for both the PDX-110 and PDX-322 samples. Genes expressed in fewer than three cells, and cells expressing fewer than 200 genes, were disregarded and a matrix of log2(1 + count per 100 kb) was input to inferCNV. Gene coordinates were determined from gene symbols using a query of Ensembl via BioMart. The inferCNV script was called with default settings (a cutoff value of 1, a noise filter of 0.2, and visualization thresholds of ±1).

### Cellular barcoding computational analysis

Raw sequencing data was preprocessed using the R package edgeR^50^ as previously^51^. In brief, reads were split according to sample index, and barcodes associated with each sample were recorded in a barcode-count matrix. To ensure high quality of the data, samples with <8000 reads, as well as samples with less than two technical replicates having over 8000 reads were removed from downstream analysis. To filter out non-specific PCR amplification, barcodes detected in less than two technical replicates per samples were discarded. Technical replicates were pooled (except for Supplementary Figure 1H) by adding the reads in each replicate and barcode, and normalized to one (i.e. such that the sum over all barcodes per sample equaled one). If technical replicates from the same sample were processed in different sequencing runs, they were first normalized, then pooled, and normalized again, to mitigate the effects of differing sequencing library sizes. Filtered and normalized data was analyzed and visualized using the statistical computing language R, specifically the package ggplot2^52^. For determining significance in the differences of means between two conditions, the Welch Two Sample *t*-test was performed on log-transformed values, and *p*-values <0.05 were considered statistically significant.

Sampling of barcodes from primary tumor was performed with replacement, with the probability of a given barcode being sampled proportional to its measured frequency in the primary tumor. The sample size equalled the number of cells sorted by FACS for a given tissue.

For simulating growth of barcoded tumor cells in three dimensions, the C++ code developed in Waclaw, B. et al.^34^ was run using the original parameters for growth and migration, but was adapted to include the additional barcode information. To mimic our experimental data, simulations were initiated with 200 barcoded tumor-initiating cells, and outcomes were rendered using the PyMol software. Tumor biomass representation in a distal tissue corresponds to sum of frequencies in primary tumor of barcoded clones that are shared between primary and the distal tissue.

### Reporting summary

Further information on experimental design is available in the Nature Research Reporting Summary linked to this article.

## Supplementary information

Supplementary Information

Reporting Summary

## Data Availability

The scRNA-seq reads counts used for inferred copy number analyses are available on GEO as series GSE123926. Barcoding datasets generated during the current study are available from the corresponding authors on reasonable request.
