# Publisher Correction: Barcoding reveals complex clonal behavior in patient-derived xenografts of metastatic triple negative breast cancer

**DOI:** 10.1038/s41467-019-09916-1

**Published:** 2019-04-24

**Authors:** D. Merino, T. S. Weber, A. Serrano, F. Vaillant, K. Liu, B. Pal, L. Di Stefano, J. Schreuder, D. Lin, Y. Chen, M. L. Asselin-Labat, T. N. Schumacher, D. Cameron, G. K. Smyth, A. T. Papenfuss, G. J. Lindeman, J. E. Visvader, S. H. Naik

**Affiliations:** 1 ACRF Stem Cells and Cancer Division, The Walter and Eliza Hall Institute of Medical Research, Parkville, VIC 3052 Australia; 2 Department of Medical Biology, The University of Melbourne, Melbourne, VIC 3010 Australia; 3 Olivia Newton-John Cancer Research Institute, Heidelberg, VIC 3084 Australia; 4 School of Cancer Medicine, La Trobe University, Bundoora, VIC 3086 Australia; 5 Molecular Medicine Division, The Walter and Eliza Hall Institute of Medical Research, Parkville, VIC 3052 Australia; 6 Bioinformatics Division, The Walter and Eliza Hall Institute of Medical Research, Parkville, VIC 3052 Australia; 7 Immunology Division, The Walter and Eliza Hall Institute of Medical Research, Parkville, VIC 3052 Australia; 8 Division of Molecular Oncology & Immunology, Netherlands Cancer Institute, Amsterdam, 1066 CX The Netherlands; 9 School of Mathematics and Statistics, The University of Melbourne, Melbourne, VIC 3010 Australia; 10 Peter MacCallum Cancer Centre, Melbourne, VIC 3000 Australia; 11 Sir Peter MacCallum Department of Oncology, University of Melbourne, Melbourne, VIC 3010 Australia; 12 Department of Medical Oncology, The Peter MacCallum Cancer Centre, Melbourne, VIC 3000 Australia; 13 Department of Medicine, The University of Melbourne, Melbourne, VIC 3010 Australia; 14 Parkville Familial Cancer Centre, The Royal Melbourne Hospital and Peter MacCallum Cancer Centre, Parkville, VIC 3050 Australia

**Keywords:** Genetic techniques, Breast cancer, Tumour heterogeneity

Correction to: *Nature Communications* 10.1038/s41467-019-08595-2, published online 15 February 2019

The original version of this Article contained an error in Fig. 4. In the left histogram of the right panel of Fig. 4d, several data points were inadvertently deleted from the histogram during the production process. This error has been corrected in both the PDF and HTML versions of the Article. The original, incorrect version of Fig. 4 is presented below as Fig. 1.

**Figure 1:**
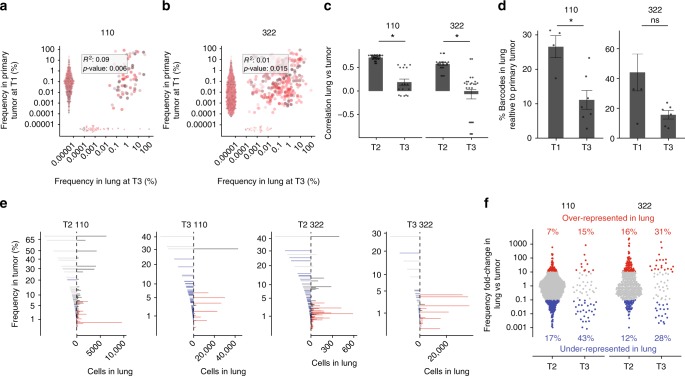
Fig. 1

