# Supplementary material for: Barcoding reveals complex clonal behavior in patient-derived xenografts of metastatic triple negative breast cancer
Source: Nat Commun. 2019 Feb 15;10:766. doi: 10.1038/s41467-019-08595-2 (PMC6377663; doi:10.1038/s41467-019-08595-2)
Supplement: Supplementary file 1 — Supplementary Information [file 41467_2019_8595_MOESM1_ESM.pdf]

Supplementary Information

Barcoding reveals complex clonal behavior in patient-derived xenografts of metastatic triple negative breast cancer

Merino, Weber et al.

Supplementary Table 1. Patient number, presence of a mutation in the *BRCA1* and *BRCA2* genes, metastasis and outcome for patient 110, 322 and 744

Patient Number	Mutation status	Metastasis	Survival
**110**	BRCA1	Brain, lung , bone (spine) metastases	1.8 year
**322**		Lymph nodes and bilateral adrenal metastasis	Alive (> 9.5 years)
**744**		LN, metastasis in liver, chest wall and bones	6.5 years

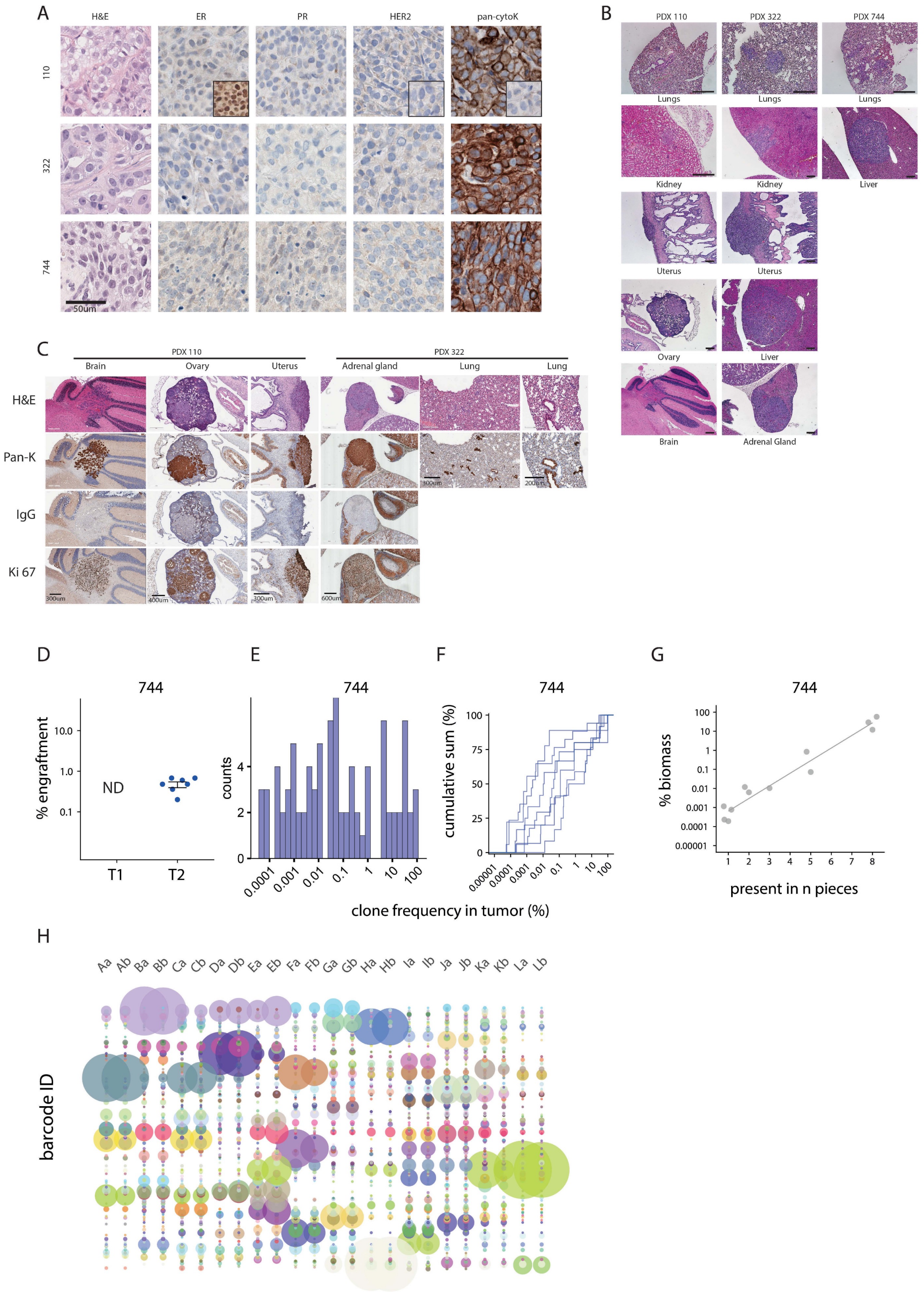
A
H&E
ER
PR
HER2
pan-cytoK
110
322
744
50um
B
PDX 110
PDX 322
PDX 744
Lungs
Lungs
Lungs
Kidney
Kidney
Liver
Uterus
Uterus
Ovary
Liver
Brain
Adrenal Gland
C
PDX 110
PDX 322
Brain
Ovary
Uterus
Adrenal gland
Lung
Lung
H&E
Pan-K
IgG
Ki 67
300um
400um
300um
600um
300um
200um
D
744
% engraftment
10.0
1.0
0.1
ND
T1
T2
E
744
counts
4
2
0
0.0001
0.001
0.01
0.1
1
10
100
F
744
cumulative sum (%)
100
75
50
25
0
0.00001
0.0001
0.001
0.01
0.1
1
10
100
clone frequency in tumor (%)
G
744
% biomass
100
10
1
0.1
0.01
0.001
0.0001
0.00001
1
2
3
4
5
6
7
8
present in n pieces
H
Aa
Ab
Ba
Bb
Ca
Cb
Da
Db
Ea
Eb
Fa
Fb
Ga
Gb
Ha
Hb
Ia
Ib
Ja
Jb
Ka
Kb
La
Lb
barcode ID

Supplementary Figure 1. Characterization of PDX-110, -322 and -744, related to Figure 1 and 2.

A) Characterization of primary tumors from PDX-110, -322 and -744, by H&E, ER, PR, HER2 and pan-CK (pan-cytokeratin) staining. Inserts: positive controls for ER and a negative control for HER2 expression and pan-CK. Scales bars, 50 µm. **B)** Histology sections of metastases from PDX-110, PDX-322 and PDX-744 in different tissues. Scale bar, 200 µm. **C)** H&E, pan-CK, IgG and KI67 staining on metastasis from different organs. Scales bars, as indicated in the figure. **D)** Engraftment efficiencies of PDX-744 assessed at T1 and T2. ND: not determined. **E)** Frequency distribution of clones at T2 pooled 7 mice for PDX-744. **F)** Cumulative size distribution of clones in primary tumor for PDX-744 (n=7 mice from 2 independent experiments). **G)** Relationship of % biomass and the number of pieces a clone is detected for PDX-744. **H)** Bubble plot of clonal relationships between tumor pieces as represented in Figure 1F, showing side-by-side the two replicates per tumor piece. This result was representative of multiple tumors from multiple independent experiments.

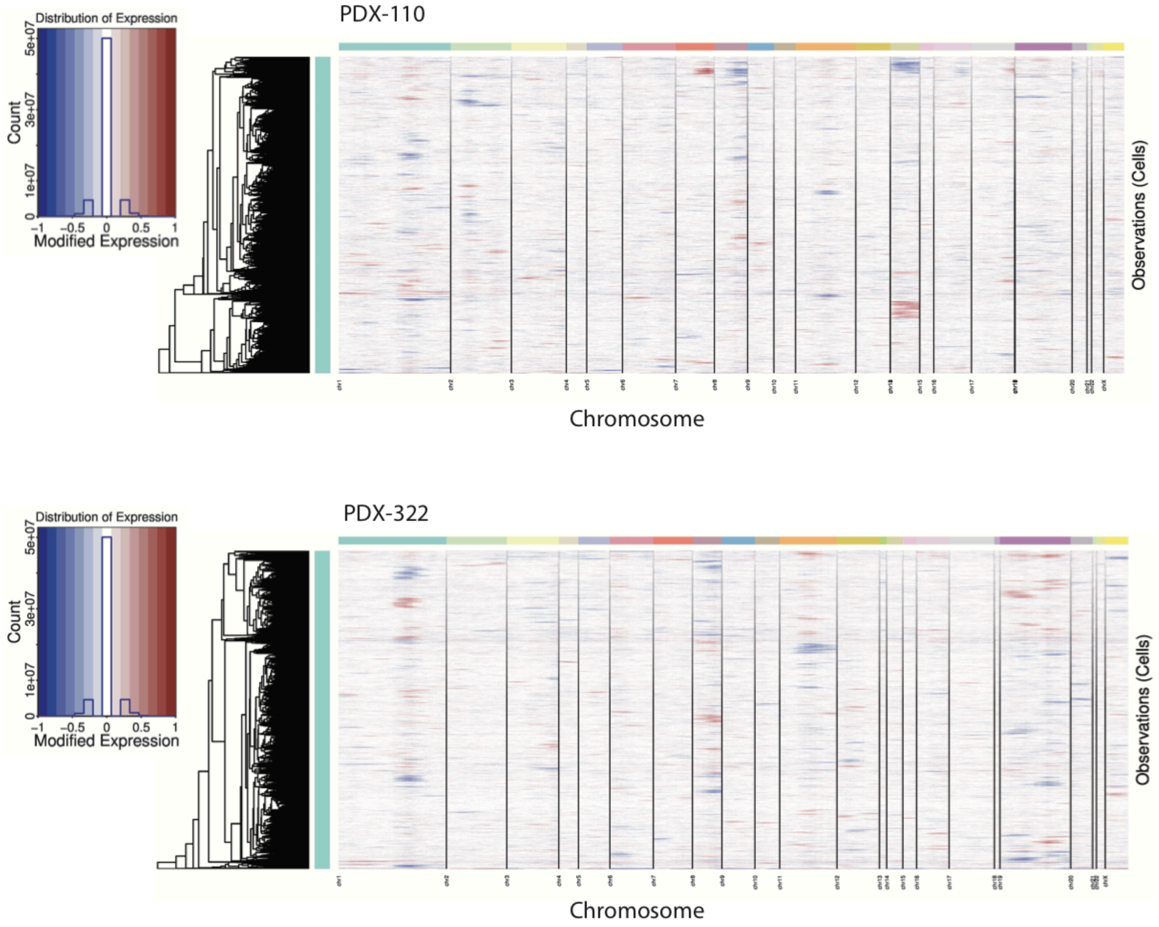

Supplementary Figure 2. Single Cell Copy Number Variation in PDX-110 and PDX-322

Single cell suspensions of early passage xenografts PDX-110 and -322 were run on a 10X Chromium platform using the 3’ single cell protocol. Copy number variations within single cells were inferred using the InferCNV algorithm (see Methods). Cells are in rows, Chromosomes in columns, with color indicative of inferred CNVs.

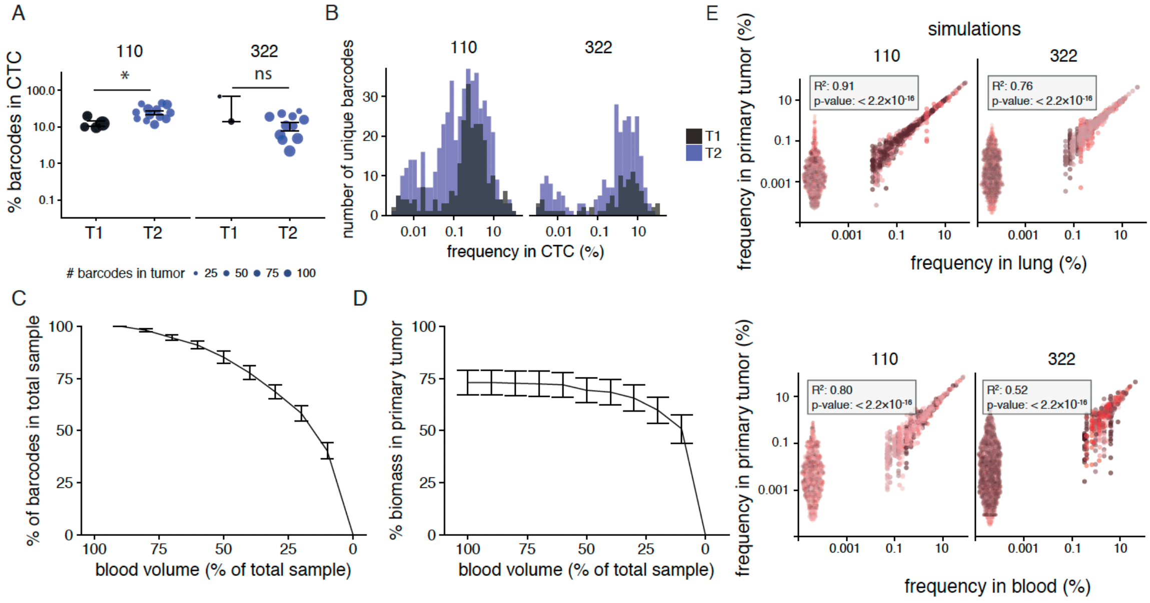

Supplementary Figure 3. Analysis of shedders, related to Figure 3.

A) Number of clones detected at T1 and T2 for PDX-110 and PDX-322. Size of dots is proportional to the total number of clones in primary tumor. Welch two-sample t-test, * p <0.05. ns = non-significant. **B)** Frequency distribution of clones in CTCs at T1 and T2 pooled over several mice (PDX-110: 10 mice at T1, 8 mice at T2, from 2 independent experiments and PDX-322: 13 mice at T1, 11 mice at T2 from 3 independent experiments). **C)** Simulation of number of clones in blood and lung at T2 estimated from random sampling. **D)** Percentage of detected barcodes in total blood sample as a function of the percentage of total blood sample. **E)** Percentage biomass in primary tumor of detected barcodes in blood as a fur of the percentage of total blood sample.

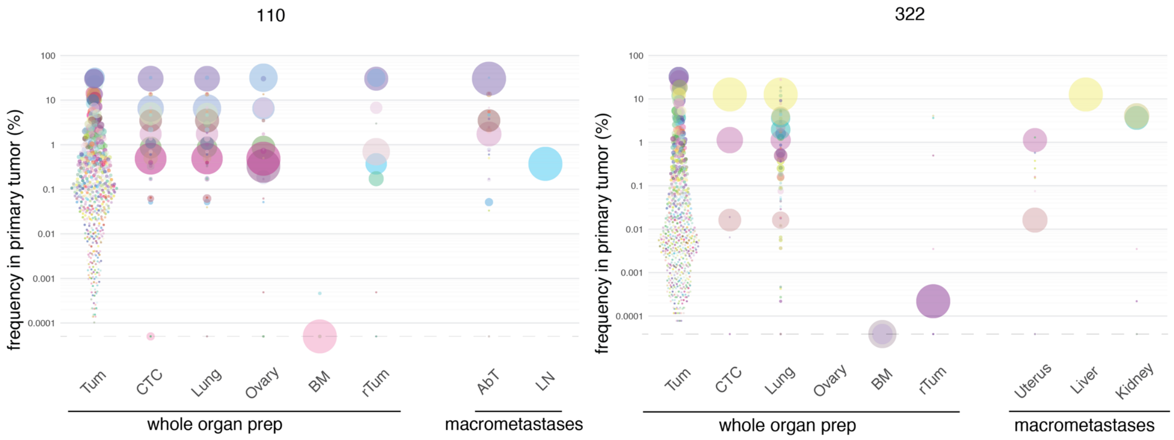

Supplementary Figure 4. Analysis of seeders, related to Figure 5.

Bubble plot pooled over several mice plot of clonal relationships between primary tumor and several metastatic sites of PDX-110 and PDX-322: 3 mice. Relapse tumor is denoted by rTum, abdominal tumor by AbT, and bone marrow by BM.

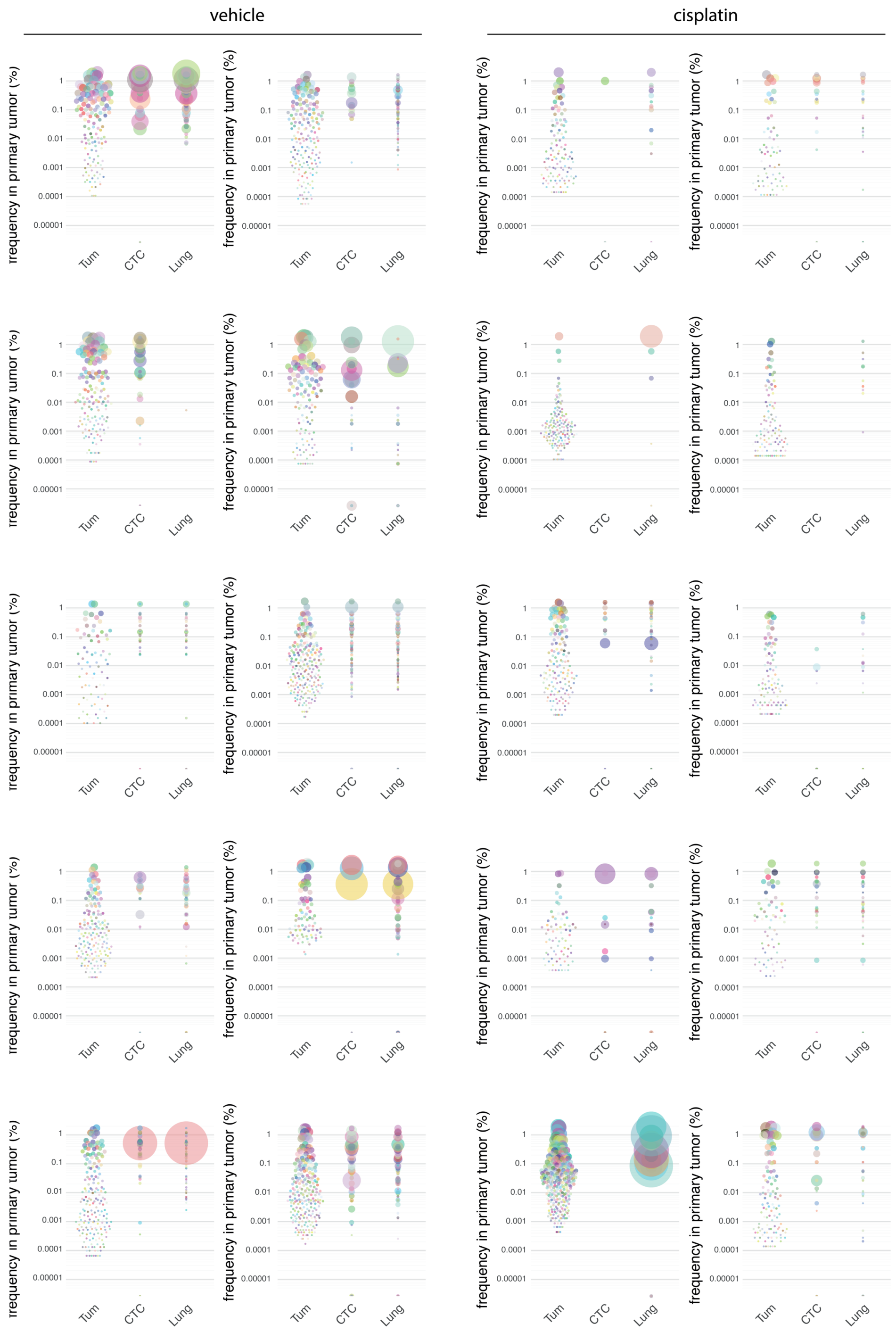

Supplementary Figure 5. Effect of chemotherapy on tumor heterogeneity, related to Figure 6.

Clonal relationships between primary tumor, blood and lung in vehicle and cisplatin-treated mice. Each graph corresponds to one mouse, each color represents a barcode.
