# Supplementary material for: Barcoding reveals complex clonal behavior in patient-derived xenografts of metastatic triple negative breast cancer
Source: Nat Commun. 2019 Feb 15;10:766. doi: 10.1038/s41467-019-08595-2 (PMC6377663; doi:10.1038/s41467-019-08595-2)
Supplement: Supplementary file 2 — Reporting Summary [file 41467_2019_8595_MOESM2_ESM.pdf]

nature research

Corresponding author(s): Delphine Merino, Jane Visvader, Shalin Naik

Last updated by author(s): Dec 17, 2018

Reporting Summary

Nature Research wishes to improve the reproducibility of the work that we publish. This form provides structure for consistency and transparency in reporting. For further information on Nature Research policies, see Authors & Referees and the Editorial Policy Checklist.

Statistics

For all statistical analyses, confirm that the following items are present in the figure legend, table legend, main text, or Methods section.

n/a	Confirmed	
☐	☒	The exact sample size (*n*) for each experimental group/condition, given as a discrete number and unit of measurement
☐	☒	A statement on whether measurements were taken from distinct samples or whether the same sample was measured repeatedly
☐	☒	The statistical test(s) used AND whether they are one- or two-sided<br>*Only common tests should be described solely by name; describe more complex techniques in the Methods section.*
☐	☒	A description of all covariates tested
☐	☒	A description of any assumptions or corrections, such as tests of normality and adjustment for multiple comparisons
☐	☒	A full description of the statistical parameters including central tendency (e.g. means) or other basic estimates (e.g. regression coefficient) AND variation (e.g. standard deviation) or associated estimates of uncertainty (e.g. confidence intervals)
☐	☒	For null hypothesis testing, the test statistic (e.g. *F*, *t*, *r*) with confidence intervals, effect sizes, degrees of freedom and *P* value noted<br>*Give P values as exact values whenever suitable.*
☒	☐	For Bayesian analysis, information on the choice of priors and Markov chain Monte Carlo settings
☒	☐	For hierarchical and complex designs, identification of the appropriate level for tests and full reporting of outcomes
☒	☐	Estimates of effect sizes (e.g. Cohen's *d*, Pearson's *r*), indicating how they were calculated

Our web collection on statistics for biologists contains articles on many of the points above.

Software and code

Policy information about availability of computer code

Data collection	bcl2fastq v2.19.1.403 (Illumina) is available from http://support.illumina.com
Data analysis	inferCNV v0.3 is available from https://github.com/broadinstitute/inferCNV<br>Cell Ranger v2.0.0 (10X Genomics) is available from https://support.10xgenomics.com

For manuscripts utilizing custom algorithms or software that are central to the research but not yet described in published literature, software must be made available to editors/reviewers. We strongly encourage code deposition in a community repository (e.g. GitHub). See the Nature Research guidelines for submitting code & software for further information.

Data

Policy information about availability of data

All manuscripts must include a data availability statement. This statement should provide the following information, where applicable:

- Accession codes, unique identifiers, or web links for publicly available datasets
- A list of figures that have associated raw data
- A description of any restrictions on data availability

The scRNA-seq reads counts used for inferred copy number analyses are available on GEO as series GSE123926. Barcoding datasets generated during the current study are available from the corresponding authors on reasonable request.

Field-specific reporting

Please select the one below that is the best fit for your research. If you are not sure, read the appropriate sections before making your selection.

☒ Life sciences ☐ Behavioural & social sciences ☐ Ecological, evolutionary & environmental sciences

For a reference copy of the document with all sections, see nature.com/documents/nr-reporting-summary-flat.pdf

Life sciences study design

All studies must disclose on these points even when the disclosure is negative.

Sample size	The number of mice for each cohort was determined by the number of GFP positive cells that were obtained after infection at low MOI. The number of mice were indicated in the legend of each figure.
Data exclusions	We initially performed analysis on 3 different PDXs (322, 110 and 774) and didn't pursue the analysis of metastasis in PDX 744 as the number of barcoded clones engrafted in the primary tumor was insufficient.
Replication	The experiments were repeated as indicated in the figure legends, to ensure the reproducibility of the results.
Randomization	For the in vivo experiment using Cisplatin treatment, mice were randomized into 2 groups (Vehicle and cisplatin treated) using the Study Director software (v 3.0, studylog). For all the other experiments, all the results were included in the analysis.
Blinding	Blinding wasn't possible in the experiments, but the authors have processed a large number of samples with minor communication at the time of collection/sequencing and analysis of the samples. DM performed most of the in vivo experiments with the help of FV,KL and AS. Dm and AS performed most of the PCR and sequencing experiments, and TW analyzed the data. The authors discussed the results after analysis.

Reporting for specific materials, systems and methods

We require information from authors about some types of materials, experimental systems and methods used in many studies. Here, indicate whether each material, system or method listed is relevant to your study. If you are not sure if a list item applies to your research, read the appropriate section before selecting a response.

Materials & experimental systems

n/a	Involved in the study
☐	☒ Antibodies
☒	☐ Eukaryotic cell lines
☒	☐ Palaeontology
☐	☒ Animals and other organisms
☐	☒ Human research participants
☒	☐ Clinical data

Methods

n/a	Involved in the study
☒	☐ ChIP-seq
☒	☐ Flow cytometry
☒	☐ MRI-based neuroimaging

Antibodies

Antibodies used

Antibodies against ER (NCL-L-ER-6F11, Novocastra), PR (NCL-L-PGR-312, Novocastra), HER2 (SP3, Spring Bioscience) or pan-cytokeratin (DAKO, Clone AE1/AE3) were used

Validation

All these antibodies are commonly used at the Royal Melbourne Hospital for the diagnosis of patients with breast cancer.
Many references can also be find on pubmed. Here are some examples (for IHC):
- ER antibody:
-- Soares M,Ribeiro R,Najmudin S,Gameiro A,Rodrigues R,Cardoso F,Ferreira F, Oncotarget
-- Corrêa NC,Kuasne H,Faria JA,Seixas CC,Santos IG,Abreu FB,Nonogaki S,Rocha RM,Aparecida Borges Silva G,Gobbi H,Rogatto SR,Goes AM,Gomes DA, Oncology Reports
-- Goyal HO,Braden TD,Williams CS,Dalvi P,Mansour MM,Mansour M,Williams JW,Bartol FF,Wiley AA,Birch L,Prins GS, Biology of Reproduction
-- Herbert M,Sandbank J,Liokumovich P,Yanai O,Pappo I,Karni T,Segal M, Histopathology

- PR antibody:
-- Ugras N, Yerci O, Coşkun S, Ocakoglu G, Sarkut P, Dündar H., Kaohsiung J Med Sci.
-- Winczura P, SosiÅ„ska Mielcarek K, Duchnowska R, Badzio A, Lakomy J, Majewska H, et al., Pathol Oncol Res.
-- Jones M, Fusi L, Higham J, Abdel Hafiz H, Horwitz K, Lam E, et al., Proc Natl Acad Sci U S A.
-- Bellance C, Khan J, Meduri G, Guiochon Mantel A, Lombes M, Loosfelt H.. Mol Biol Cell. 2

HER2:
-- Tian M & Schiemann WP, J Cancer Metastasis Treat

-- Park SY et al. Br J Radiol

- Pan-cytokeratin:
-- Lin, W., Yip, Y. L., et al. Nature Comm
-- Flammini, L., Mantelli, L., et al. Veterinary Medicine and Science
-- Kondrashova, O., Topp, M., et al., Nature Comm

Animals and other organisms

Policy information about studies involving animals; ARRIVE guidelines recommended for reporting animal research

Laboratory animals	3 to 4-week-old NOD-SCID-IL2Rγc–/– female mice
Wild animals	N/A
Field-collected samples	N/A
Ethics oversight	All animals were bred and maintained in the WEHI mouse facility, following the institutional guidelines. Protocols were approved by the WEHI Animal Ethics Committee.

Note that full information on the approval of the study protocol must also be provided in the manuscript.

Human research participants

Policy information about studies involving human research participants

Population characteristics	Breast cancer primary tumors were collected after consentment of the patients through the Royal Melbourne Hospital Tissue Bank and the Victorian Cancer Biobank with relevant institutional review board approval. The 3 patients were women, age at diagnosis: 35, 36 and 67. Tumours were collected prior treatment. The patient details have been de-identified.
Recruitment	These patients were recruited as they presented with resectable breast cancer tumors. They consented to give their tumors for research at the time of surgery.<br>In this study, we selected these patient derived xenografts (amongst others) as they were coming from patients with triple negative breast cancer, and based on their ability to metastasize to multiple organs.
Ethics oversight	Human ethics were approved by the Walter and Eliza Hall Institute (WEHI) Human Research Ethics Committee.

Note that full information on the approval of the study protocol must also be provided in the manuscript.
